# The effect of different cardiovascular risk presentation formats on intentions, understanding and emotional affect: a randomised controlled trial using a web-based risk formatter (protocol)

**DOI:** 10.1186/1472-6947-10-41

**Published:** 2010-07-30

**Authors:** Cherry-Ann Waldron, John Gallacher, Trudy van der Weijden, Robert Newcombe, Glyn Elwyn

**Affiliations:** 1 Department of Primary Care and Public Health, School of Medicine, Cardiff University, Cardiff, UK; 2 CAPHRI, School of Public Health and Primary Care, Department of General Practice, Maastricht University, Maastricht, The Netherlands

## Abstract

**Background:**

The future risk of heart disease can be predicted with increasing precision. However, more research is needed into how this risk is conveyed and presented. The aim of this study is to compare the effects of presenting cardiovascular risk in different formats on individuals' intention to change behaviour to reduce risk, understanding of risk information and emotional affect.

**Methods/design:**

A randomised controlled trial comprising four arms, with a between subjects design will be performed. There will be two intervention groups and two control groups. The first control comprises a pre-intervention questionnaire and presents risk in a bar graph format. The second control presents risk in a bar graph format without pre-intervention questionnaire. These two control groups are to account for the potential Hawthorne effect of thinking about cardiovascular risk before viewing actual risk. The two intervention groups comprise presenting risk in either a pictogram or metonym format (image depicting seriousness of having a myocardial infarction). 800 individuals' aged between 45 and 64 years, who have not been previously diagnosed with heart disease and have access to a computer with internet, will be given a link to a website comprising a risk calculator and electronic questionnaires. 10-year risk of having a coronary heart disease event will be assessed and presented in one of the three formats. A post-intervention questionnaire will be completed after viewing the risk format. Main outcome measures are (i) intention to change behaviour, (ii) understanding of risk information, (iii) emotional affect and (iv) worry about future heart disease. Secondary outcomes are the sub-components of the theory of planned behaviour: attitudes, perceived behavioural control and subjective norms.

**Discussion:**

Having reviewed the literature, we are not aware of any other studies which have used the assessment of actual risk, in a trial to compare different graphical cardiovascular risk presentation formats. This trial will provide data about which graphical cardiovascular risk presentation format is most effective in encouraging behaviour change to reduce cardiovascular risk.

**Trial registration:**

Current Controlled Trials ISRCTN91319318

## Background

The risk of heart disease can be predicted with increasing with precision, with the development of algorithms such as Framingham, SCORE and QRISK2 [1-3]. Less is known about how to portray and communicate cardiovascular risk in ways that motivate people to modify their lifestyle to reduce this risk. However, recent research on the effects of presenting coronary risk information found that the presentation of global coronary heart disease (CHD) risk estimates can improve the accuracy of risk perceptions and increase intention to initiate prevention strategies [4]. A systematic review on the effects of different interventions used to communicate cardiovascular risk [5], found that studies comparing interventions for cardiovascular risk presentation have been heterogeneous in design, and that many have been of low methodological quality. Very few studies assessed patients' actual risk. The majority were analogue studies where individuals were asked to imagine a hypothetical risk. There was no consistency in which presentation formats were used (percentages and natural frequencies were the most commonly assessed), and only a small number of studies used graphical representations (mainly bar graphs and pictograms). A wide range of outcome measures were assessed, including changes in risk and risk factors, intention to change behaviour and acceptance rates of treatment. The lack of coherent research, and need for methodologically sound trials provides the basis for this proposed trial. The purpose of this trial is to assess cardiovascular risk communication strategies and their impact on preventative behavioural intentions.

At least 80 percent of heart disease, stroke and type 2 diabetes are thought to be attributed to the modifiable risk factors of poor diet, lack of physical activity and tobacco use [6]. Therefore, lifestyle and behaviour change is important in order to reduce the impact of these factors and decrease the incidence rate of heart disease in the population. Informing patients of their future risk is the first step in helping them make decisions about reducing their risk. However, the way this risk information is formatted and framed can influence understanding, perceptions and behaviours [7-10]. Difficulties in communicating cardiovascular risk arise due to the interaction of many variables. Most research has focused on epidemiological precision rather than on how to motivate behaviour change. It has also overlooked the major contribution to risk arising from age (an unmodifiable risk variable) and the difficulty people have in considering risk over long time horizons, such as 10 years spans [11].

Although previous research has compared different graphical risk presentation formats relating to conditions such as diabetes and hereditary breast cancer [12-15]; studies have yet to compare graphical cardiovascular risk presentation formats with each other, especially when assessing and presenting patients' actual risk [5].

There are numerous, commonly used theoretical approaches to health behaviour and behaviour change [16-19]. However, of particular interest is the Theory of Planned Behaviour (TPB) [20]. This theory postulates that behaviour is determined by a small number of factors, namely attitudes, subjective norms and perceived behavioural control. It has empirical support for predicting a wide range of behaviours [21,22]. It predicts intention, which is generally regarded as a strong predictor of behaviour, as people tend to engage in behaviours that they plan to perform [23]. In instances where it is not feasible to measure actual behaviours, intention can be an adequate proxy.

Models are unable to account for every factor that influences behaviour. Most health behaviour theories fail to consider emotion or affect in the form of 'feelings' as opposed to 'affective judgements' [24]. Emotions are important when considering behaviour relating to the reduction of cardiovascular risk, as they have been shown to influence perceptions of risk [25,26] which in turn, can affect health-related behaviour [27]. In our systematic review on cardiovascular risk communication [5], emotional responses to cardiovascular risk were only addressed in analogue studies requiring individuals to imagine a hypothetical risk profile. There is currently little information on the impact of emotions when people are asked to consider their actual cardiovascular risk.

'Worry' is extremely relevant when thinking about one's future risk of heart disease. It has been associated with risk perception and is referred to as cognition 'coloured by affect' [28,29]. Some suggest that worry contains an appraisal of risk elements (such as likelihood and loss) [30] and is not necessarily maladaptive. Specifically, previous research has found that worry positively predicts behavioural intentions [31]. When at high levels, worry can lead to the uptake of screening behaviour [32] and has found to be the strongest predictor of contemplation to quit smoking [33]. However, evidence has also been found for an inverted-U or curvilinear relationship between worry and consequent behaviour [34]. Too much worry can lead to the activation of defensive mechanisms, where incoming information is ignored or distorted [35]. When communicating cardiovascular risk, we do not know how much worry is beneficial and would lead to increasing an individual's motivation to reduce risk versus denial. At what level does worry induce a positive intention to reduce risk, and is there an optimum level before the risk communication process becomes inhibitory?

Patients' understanding of their own cardiovascular risk is generally poor to the point of being non-existent. In addition, there is evidence to show that the data presented in cardiovascular risk prediction tools is often misunderstood [26,36]. This can inhibit people from making informed decisions regarding their health and behaviour. There is as yet no consensus as to which format is most effective in terms of facilitating patient understanding of their risk information [37]; and also, what the most appropriate way to measure understanding actually is. It is argued that current attempts used in the communication of cardiovascular risk, such as recall, self-reported confidence in understanding and perceived difficulty in understanding are not suitable methods; as repetition and personal judgements do not indicate that individuals' have derived the correct meaning and possess a true understanding [5].

A prerequisite of understanding health related risk information is adequate numeracy and literacy skills. These are poor in many adults, leading to difficulty with simple decimal places and ratio concepts (including fractions, proportions and probabilities) [38]. It has been documented that smokers with lower literacy skills, are less likely to understand their risk of heart disease and stroke [39]. This may be because interpreting risk information involves a hierarchy of skills ranging from calculation, inferences and interpreting tables and charts, which is problematic for those with lower levels of numeracy [40]. Therefore, an important question is: can understanding of risk information be improved? Are there alternative ways of presenting cardiovascular risk to individuals' that are not numerically-based, precise estimates, but more qualitative, gist representations? These are arguably what are most required, as they are used when 'interpreting' the given risk information [41].

One contender for representing gist information is the concept of a metonym. This is a type of metaphor and involves part and whole relations and associations. It is a word for a part of something, used to refer to the whole entity; or the whole is referred to in terms of something associated with it [42]. An example would be representing heart disease by using the concept of a myocardial infarction. Metonyms are important to everyday life as their concepts structure thoughts, attitudes and actions, as well as language [43]. Using a metonym to present future risk of a disease could be a way of improving affective forecasting, as people are not good at predicting the future [44]. It is a striking symbolisation what the disease encompasses, rather than an abstract numerical value. As far as we are aware, there are no existing studies that have used the metonym concept to present risk information.

The concept defined as correlational validity by Ubel [45], can be used to test whether individuals are applying their knowledge and understanding rationally. For example, men and women at high risk of heart disease should be more willing to take statins or blood pressure lowering drugs, than those at moderate or low risk. Therefore, it follows that if high risk individuals understand the risk information presented to them, they should be more likely to have greater intentions to change behaviour to reduce risk and vice versa.

## Aims and Objectives

The overall aim of this trial is to compare the effects of different graphical cardiovascular risk presentation formats on individuals' intention to reduce risk, understanding of risk information, emotional affect and worry about future heart disease, using a web-based risk calculator.

The primary objectives of this study are:

• To assess which format leads to the greatest intention to change behaviour.

• To determine which format best facilitates understanding of risk information.

• To analyse which format alters emotional affect.

• To assess which format induces worry about future heart disease the most.

• To examine the correlational validity between intention to change behaviour, understanding of risk and worry about future heart risk. To find out if understanding results in more appropriate intentions regarding cardiovascular risk and what level of worry increases intention to change behaviour.

• To determine whether intention to change behaviour, understanding of risk, and emotional affect are mediated by a person's risk category.

The secondary objectives of this study are:

• To examine the existence of the Hawthorne effect using two control groups.

• To analyse within group changes between pre and post-intervention responses in the group who completed both questionnaires.

• To evaluate the use of the internet-provided risk formatter (process evaluation), including analysis of web-logs.

• To assess the TPB's efficacy to predict intention to change behaviour to reduce future heart risk.

## Methods/Design

### i) Design

A randomised controlled trial (RCT), with a between-subjects design, will be used to compare the effect of each presentation format on the specified outcomes. There will be four conditions in total, comprising two intervention groups and two control groups. This is to address the possibility of the Hawthorne effect [46] of the four groups and the effect of thinking about cardiovascular risk before viewing actual risk.

### ii) Setting

The trial will be conducted remotely from any location with access to a computer and the internet. This places no time or locality constraints on the respondents, as they can participate at their convenience.

### iii) Participants

Respondents are eligible for inclusion in the trial if aged between 45 and 64, and have not been previously diagnosed with cardiovascular disease. This is because the risk calculator algorithm is unsuitable for use in a population of those with existing heart disease due to an underestimation of risk. However, those with hypertension, hypercholesterolemia and diabetes are still eligible. Respondents must also have access to a computer with the internet, have adequate IT skills and be able to read English.

### iv) Recruitment

Respondents will be invited to take part in the study using a number of methods. In order of implementation and preference, these methods are: emails to educational institutions, co-operation with large organisations where the workforce has access to a computer; social networking websites (such as *Facebook) *and advertisements in local newspapers. The study will also be advertised on posters and pocket sized cards.

### v) Intervention and comparisons

A website comprising a cardiovascular risk formatter and questionnaires has been developed. The purpose of this tool is to enable the different risk presentation formats to be randomly assigned to respondents, creating a platform to measure the outcomes of interest. It uses the Personal Heart Score [47] which assesses 10-year risk of having a coronary heart disease (CHD) event (myocardial infarction, fatal CHD, or cardiac procedure). It uses self-reported, non-laboratory measurements such as age, gender, previous diagnosis of hypertension, hypercholesterolemia or diabetes, smoking status, family history of premature CHD (e.g. a parent who was under the age of 50 when they were told by their GP/Physician that they had a heart attack), level of physical activity (e.g. exercising or playing sport in leisure time) and body mass index. A point scoring system categorises risk into three groups (low risk < 10%; intermediate risk 10-20%; high risk > 20%). It is recognised that other algorithms such as Framingham Risk Score, SCORE or QRISK2 [1-3], provide a more precise risk estimation, especially if they include physiological measurements such as blood pressure. However, it is believed that the Personal Heart Score is most appropriate for the purpose of this study as it provides an estimation of risk level, which can easily be presented in different formats to enable a head-to-head comparison. More importantly, it enables assessment of individuals who have not thought about their cardiovascular risk before and are unaware that they may be at high risk; most of whom are unlikely to have visited a health professional to undergo formal clinical assessment. The website recommends that concerned respondents visit their GP for more formal clinical investigation and before under taking lifestyle changes, links to useful websites such as the British Heart Foundation will also be provided.

**Figure 1:**
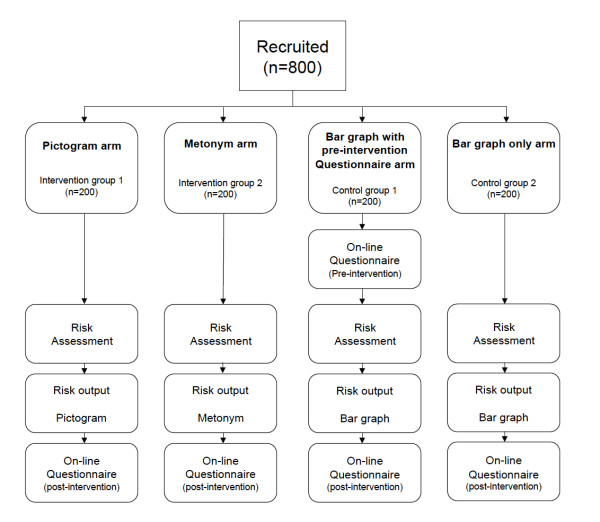
**Flowchart of the RCT with intervention and control groups**.

Before respondents can proceed, they will be given brief details about the study, asked to indicate their informed consent electronically and will be assessed for eligibility. The computer will then randomise the respondent into one of the four arms, ensuring allocation concealment (see Figure 1). These comprise a bar graph with pre-intervention questionnaire (control group 1), bar graph only (control group 2), pictogram (intervention group 1) or metonym (intervention group 2). Following the risk assessment, all respondents will be given their risk category (low, moderate or high) and the corresponding percentage figure (< 10%, 10-20% or > 20%). The main comparators will be the accompanying graphical risk presentation formats (bar graph, pictogram and metonym).

The bar graph format to be used in the two control groups consists of vertical bar graph depicting percentages. This will be animated (growing upwards) to demonstrate the wide confidence intervals of the risk categories in the Personal Risk Score [47]. A bar graph has been chosen as it is the standard presentation format commonly used in current risk prediction tools. A pictogram of 100 hearts depicting natural frequencies will be used in intervention group 1. Research shows that these formats are better understood by patients, natural frequencies intuitively offer more insight than other formats [9,10] and pictograms help the viewer see the risk in context and facilitate accurate judgements of probability [48,49]. Again, this format will be animated, highlighting each affected heart in turn, to account for the range of numbers affected in the risk category. A metonym format will be used in intervention group 2. This will depict the seriousness of an emergency admission for a myocardial infarction. This has been chosen as heart disease is generally associated with having a myocardial infarction [50]. An image demonstrating healthy longevity will be shown to those in the low risk category; those at moderate risk will be shown an ambulance traveling towards a person's house, and a person being defibrillated will be shown to the high risk category.

To assess changes in emotional affect and worry, all respondents will have these measured at baseline during the risk assessment. Those in the bar graph and pre-intervention questionnaire group (control group 1) will also complete a partially parallel version of the post-intervention questionnaire. This is to address the Hawthorne effect of the four groups, and compare those who are asked to think about their cardiovascular risk and their prior intentions to reduce this, against those who are not. However, to keep the total number of items to a minimum, the focus is on reducing overall cardiovascular risk, instead of specific behaviours that lead to risk reduction. All respondents will view the risk in the format that they have been randomly assigned to and complete the post-intervention questionnaire.

There will be two main comparisons:

1. (a) Bar graph only v. Pictogram

(b) Bar graph only v. Metonym.

This will enable a head-to-head comparison of the outcomes resulting from the different risk presentation formats.

2. Bar graph and pre-intervention questionnaire v. Bar graph only. Responses from viewing the bar graph and completing the baseline questionnaire will be compared with those from viewing the bar graph only. Additionally, within group changes between baseline and post-intervention questionnaires will be analysed in the group who completed both questionnaires (control group 1).

### vi) Outcome Assessment

Outcomes will be assessed by means of a self-complete on-line questionnaire integrated into the web-based formatter. Reliability measures, such as Cronbach's alpha for internal reliability, will be carried out on the questionnaire items at the piloting stage of this trial. Items not meeting the reliability requirements will be eliminated from the final questionnaire. To address possible response bias occurring from fatigue, items measuring the different outcomes and components of the TPB will be mixed up in the questionnaire (as recommended by Ajzen [51]).

The primary outcome measures are:

(i) *Intention to change behaviour- *Items relating to cardiovascular risk reduction were developed using guidance from a manual for constructing questionnaires based on the TPB [52]. This manual was chosen as it has been widely used in previous research that has required TPB questionnaire development [53-55]. It also provides a way of measuring the TPB contrasts directly, reducing the number of items and thus, keeping the cognitive demand of respondents to a minimum. The questionnaire items comprise three risk reducing options of: smoking cessation, exercising more and losing weight. These are the three modifiable risk factors assessed by the Personal Heart Score. The relevance of these risk reducing options will be assessed. Questions relating to smoking will only be asked to those who reported that they are current smokers; adjustment will be made for this during analysis. Responses for intention to lose weight will be assessed for appropriateness (e.g. whether those who do not report this intention actually need to lose weight). Scale items such as *'I intend to exercise more' *with a 7-point Likert response options will be used. An indirect measure of intention to change behaviour will be also assessed, by examining whether individuals take the opportunity to obtain a copy of their risk output to take to their GP.

*(ii) Understanding *- Items specific to the understanding of cardiovascular risk information have been developed and will be piloted, as no suitable validated scale currently exists. These comprise absolute probability perception e.g. *'What are your chances of having heart disease in the next 10 years?' *with three pre-defined response options (low, moderate, high*)*; subjective understanding of the risk information e.g. '*What should someone in your risk category do to reduce their risk of heart disease*?' with 3 pre-defined response options (do nothing to reduce their risk, try and do a little bit to reduce their risk, do as much as they can to reduce their risk); and confidence in understanding e.g. '*How confident are you that you have understood the risk information given to you?' *with a 7 point Likert Scale to indicate level of confidence in understanding.

*(iii) Emotional affect after viewing cardiovascular risk - *The Positive and Negative Affect Schedule- Short Form (PANAS-SF) will be used [56]. This is a 10-item truncated version of the PANAS, which has been well validated and cited in over 2,000 scholarly papers [57]. It was felt that the original 20-items would be too time-consuming and cognitively demanding for respondents, possibly leading to high drop out rates. As this study is interested in respondents' changes of affect after viewing their risk output, a slight adaptation to the wording of the instructions and anchors/pole labels was made to the post-intervention scale, to make it more logical. An example of one item is: '*Thinking about yourself right now at this present moment, to what extent do you feel upset?' *with a 5-point Likert response scale anchored *'not at all' *to *'extremely'*.

*(iv) Worry about future heart disease - *one item will be used to measure this construct, in order to keep the total time needed to complete the questionnaires to a minimum. No previously developed and validated scale regarding worry about future risk of heart disease currently exists. Therefore, the item was developed using previously validated scales relating to other health conditions, such the Lerman Breast Cancer Worry Scale [58] as a guide. This item is '*After viewing your results, how worried do you feel about developing heart disease in the future?*' with '*very worried' *to '*not at all worried' *anchored on a 7-point Likert response scale.

The following secondary outcomes will also be assessed; these comprise the sub-components of the TPB [20]: attitudes, perceived behavioural control and subjective norms. Items were developed to measure the components directly according to the manual by Francis et al [52]. They will measure the three risk reducing options (smoking cessation, exercising more, losing weight). Again, relevance of the risk reducing options will be assessed, and those not applicable will be omitted from the post-intervention questionnaire.

• *Attitudes *- This comprises evaluative (evaluation using bipolar opposites), instrumental (whether the behaviour achieves something) and experiential (how it feels to perform the behaviour) items. An example is *'For me, stopping smoking would be ......' *with a 7-point Likert scale anchored '*pleasant*' to '*unpleasant*'.

• *Perceived Behavioural Control *- Items relate to either self-efficacy or the controllability of the behaviour. An example of a controllability item is '*Whether I lose weight or not is entirely up to me' *with a 7-point Likert scale to indicate the extent to which the respondent agrees with the statement. An example of a self-efficacy item is '*I am confident that I can exercise more' *with 'very *confident*' to '*not at all confident*' anchored on a 7-point Likert scale.

• *Subjective Norms - *These relate to the perceptions of significant others' preferences about whether one should or should not engage in a specific behaviour. An example is '*I feel under social pressure to lose weight' *with a 7-point Likert scale to indicate the extent to which the respondent agrees with the statement.

Other data collection comprises:

• *Respondents characteristics *(risk category, gender, age, family history of heart disease, diagnosis of hypertension, hypercholesterolemia, diabetes, smoking status, physical activity status, height and weight for BMI calculation and whether the respondent requests an electronic copy of their risk output for their GP).

• *Web logs *examining how long respondents take to complete the study and how long they spend on each page.

### vii) Sample size calculation

For simplicity, the sample size calculation is based on a comparison of means, though the analysis will recognise the ordinal nature of the data. It is hard to speculate on the difference between the groups and so the sample size is based on comparing 2 groups on the primary outcome measure which is intention to change behaviour; this will give a group size which will be used for all the groups. Recruitment will continue until 800 respondents (200 in each group) have completed the trial. The likely uptake rate is unknown and a number of the suggested recruitment methods may be needed. Based on a study that used a similar Likert Scale scoring system for a different risk context [59], the scores on intention to change behaviour within a group should have an SD of about 1.5. The total sample size in each group of 200 would then be sufficient to detect a difference of 0.5 point between two groups, with 90% power and significance value of *α = *0.05.

### viii) Analysis

The results will stored on a SQL database and fed back to the researcher via the server that hosts the website. The data will be stored on the shared drive which will be password protected and only accessible to the researcher. Data will be retrieved, coded and inputted into computer software. Microsoft Office Excel 2007 will be used for data manipulation and SPSS version 16 for the main data analyses.

The usual descriptive statistics will be presented to summarise baseline characteristics of the study sample. Continuous variables such as age and level of cardiovascular risk will be summarised using mean and SD and/or median and quartiles. Binary variables such as gender and whether the respondent requests an electronic copy of their risk output for their GP will be summarised by counts and proportions. Summary statistics will be obtained for the study population as a whole, and for the four randomised groups, without formal testing of statistical significance of any differences between them.

The main analyses of efficacy will relate to the primary outcome measures: intention to change behaviour, understanding of risk information, emotional affect and worry about future heart disease. Summary statistics for the four groups will be presented, as above. The four groups will first be compared on an equal footing using one-way ANOVA. The three selected pairwise contrasts between the specified groups, will then be constructed (e.g. bar graph only v. pictogram; bar graph only v. metonym; and bar graph and preintervention questionnaire v. bar graph only).

Several secondary analyses will be performed. For the bar graph and preintervention questionnaire group (control group 1), paired analyses will be used to assess serial changes in outcome measures between pre- and postintervention questionnaires.

A multiple regression model will be used in a sub-group analysis to look for correlations between risk category on intention to change behaviour, understanding of risk and post worry about future heart disease outcomes, to see if responses are mediated by risk category. It will also be used to assess the correlational validity between intention to change behaviour, worry about future heart disease and understanding of risk information; to determine what level of worry increases intention to change behaviour and whether understanding also results in appropriate intentions. The subcomponents of the TPB (attitudes, perceived behavioural control and subjective norms) will also be examined, to see if they sufficiently predict intention to change behaviour (in order to test the efficacy of the model in predicting cardiovascular-related behaviour change).

The direct and indirect measures of intention to change behaviour will be correlated, to see whether those who report that they intend to change their behaviour actually take the opportunity to print out their risk output to take to their GP. Furthermore, a correlation between accurate understanding of risk information and confidence in understanding will be conducted. Lastly, Independent T-tests will compare baseline and post-intervention emotional affect and worry about future heart disease scores, to determine whether scores generally decrease after viewing a particular risk presentation format, or increase, demonstrating a possible negative impact.

For all analyses, point and interval estimates will be obtained, as well as p-values. In the event of substantial departure from Gaussian distributional form, transformation of scale and/or analogous non-parametric methods will be considered.

## Discussion

This protocol provides a detailed description of a RCT designed to compare different graphical cardiovascular risk presentation formats and evaluate their effects on patient-related outcomes. The findings will inform developers of cardiovascular risk prediction tools and risk reduction interventions, providing insight into which format is most effective in encouraging behaviour change to reduce cardiovascular risk.

As far as we are aware, this will be the first RCT to assess different cardiovascular risk graphical presentation formats using actual risk assessment, rather than relying on hypothetical risk scenarios. However, a couple of limitations should be acknowledged. Firstly, a meaningful response rate will not be able to be calculated, but web-logs will give information on response trends (such as those who do not complete the study). Secondly, there is sample bias, as this study uses a self-selecting sample and is restricted to computer literature individuals. Further possible biases will be explored in the event that no difference between the risk presentation formats occurs; this is to avoid under-estimation of their effects.

Lastly, we are only able to provide respondents with a rudimentary estimation of their future 10-year risk of having a CHD event, and present them with a risk category that has wide confidence intervals and a high level of uncertainty. This is due to the use of an algorithm that uses non laboratory, self-reported information [47]; which has been chosen to increase the feasibly of data collection. Nonetheless, this will be an adequate starting point for individuals who have not thought about visiting their GP for a clinical assessment before and may not know that they are at risk.

## Competing interests

The authors declare that they have no competing interests.

## Authors' contributions

All authors contributed to the development of the research protocol. GE is principal investigator. CAW will be responsible for the management of the trial. All authors read and approved the final manuscript.

## Pre-publication history

The pre-publication history for this paper can be accessed here:

http://www.biomedcentral.com/1472-6947/10/41/prepub
